# Effects of resveratrol on renal ischemia-reperfusion injury: A systematic review and meta-analysis

**DOI:** 10.3389/fnut.2022.1064507

**Published:** 2023-01-04

**Authors:** Tian-ying Lan, Rong-liang Dun, Dong-sheng Yao, Feng Wu, Yi-ling Qian, Yuan Zhou, Tian-tian Zhan, Ming-hai Shao, Jian-dong Gao, Chen Wang

**Affiliations:** ^1^Nephrology, Shuguang Hospital, Shanghai University of Traditional Chinese Medicine, Shanghai, China; ^2^TCM Institute of Kidney Disease, Shanghai University of Traditional Chinese Medicine, Shanghai, China; ^3^Key Laboratory of Liver and Kidney Diseases, Shanghai University of Traditional Chinese Medicine, Ministry of Education, Shanghai, China; ^4^Shanghai Key Laboratory of Traditional Chinese Clinical Medicine, Shanghai University of Traditional Chinese Medicine, Shanghai, China; ^5^Urology Surgery, Yueyang Hospital of Integrated Traditional Chinese and Western Medicine Hospital, Shanghai University of Traditional Chinese Medicine, Shanghai, China

**Keywords:** meta-analysis, oxidative stress, reactive oxygen species, renal ischemia-reperfusion injury, resveratrol, systematic review

## Abstract

Renal ischemia-reperfusion (I/R) injury may lead to acute kidney injury, which is characterized by high morbidity and mortality rates. Resveratrol (RSV) can be extracted from Chinese herbs, and multiple animal experiments have demonstrated its potential for renal protection. This systematic review evaluates the protective effect of RSV against renal I/R injury in animal models. The PubMed, Embase, Web of Science, and Science Direct databases were searched for animal experiments related to RSV in renal I/R injury from their establishment to June 2022. In total, 19 studies were included with 249 animals (129 treated with RSV and 120 as controls). The pooled analysis revealed that RSV administration significantly decreased serum creatinine (SCr) levels (16 studies, *n* = 243, WMD = −58.13, 95% CI = −79.26 to −37.00, *p* < 0.00001) and blood urea nitrogen (BUN) levels (12 studies, *n* = 163, WMD = −34.37, 95% CI = −46.70 to −22.03, *p* < 0.00001) in the renal I/R injury model. The level of malondialdehyde (MDA), an oxidative stress index, was alleviated [7 studies, *n* = 106, standardized mean difference (SMD) = −6.05, 95% CI = −8.90 to −3.21, *p* < 0.0001] and antioxidant enzymes such as glutathione (GSH) (7 studies, *n* = 115, SMD = 9.25, 95% CI = 5.51–13.00, *p* < 0.00001) and catalase (CAT) (4 studies, *n* = 59, SMD = 8.69, 95% CI = 4.35–13.03, *p* < 0.0001) were increased after treatment of RSV. The subgroup analysis suggested that 5–10 mg/kg of RSV optimally protects against renal I/R injury as both the BUN and SCr levels were significantly decreased at this dosage. The protective effects of RSV against renal I/R injury might be attributed to multiple mechanisms, such as inhibiting oxidative stress, apoptosis, inflammation, fibrillation, and promoting autophagy. For a deeper understanding of the protective effects of RSV, experimental studies on animal models and large randomized controlled trials in humans are needed.

## Introduction

As a common clinical event, acute kidney injury (AKI) is an independent risk factor for death (1). It is mostly caused by dehydration, infection, and drug toxicity in communities, which acts as a silent killer. Severe AKI more commonly occurs in hospital due to certain hospital-related factors, such as major surgery, septic shock or drug toxicity. Kidney dysfunction affects almost all systems of the body, leading to multiple organ failure (2), inducing fluid and uremic toxin retention, acid-base and electrolyte disorders, ultimately compromising the balance of the internal environment. AKI resulting in structural damage may cause irreversible nephron loss, preventing the restoration of baseline glomerular filtration rate, resulting in chronic kidney disease or persistent kidney failure. The condition eventually progresses to chronic kidney disease (CKD) or even leads to end-stage renal disease (2). Elderly patients with multiple comorbidities, especially CKD, are particularly vulnerable. The global burden of AKI-related mortality has remained high over the past 50 years (3). A meta-analysis of 154 studies, including 3,585,911 people, reported an average mortality rate AKI of 23%, but up to 49.4% of patients required kidney replacement therapy (4). The complicated causes and various circumstances of AKI pose a great challenge to its prevention and treatment.

Renal ischemia is the primary pathological change of AKI. The kidney is relatively depleted from oxygen after supplying the demands of the counter-current exchange system and is particularly vulnerable to ischemia and hypoxia. Furthermore, the rapid drop of glomerular filtration rate attributed to ischemia results in further renal damage and functional decline. The restoration of blood flow triggers a “second hit,” which is characterized as an ischemia-reperfusion (I/R) injury (5). The process of renal I/R involves reactive oxygen species, lipid peroxidation, and inflammatory reactions. Endothelial cells exhibit swelling, deformation and disordered arrangement, with the disintegration of mitochondria in renal tubular epithelial cells and leucocyte-endothelial cell interaction, causing further renal fibrosis and CKD (6).

The clinical treatment of AKI mainly obeys stage-based management, while fluid control and kidney replacement therapy are still the primary modes of treatment. Kidney replacement therapy aims at mitigating life-threatening consequences, thereby preventing death from uremia. However, the initial timing, therapy intensity and other details are still debated (7). Nevertheless, no significant difference was observed in the complications and mortality between early and delayed kidney replacement therapy strategies (8). The current treatment options are relatively general and passive, earlier intervention that directly targeted to certain specific pathogenic mechanism are expected. Limited clinical means call for the development of new treatment strategies.

Resveratrol (3,4,5-trihydroxy-*trans*-stilbene, RSV) is a polyphenol belonging to the family of stilbenes and is extracted from the Chinese herb *Polygonum cuspidatum*. It is also abundant in peanuts, berries and grapes skin. Resveratrol exhibits diverse pharmacological benefits (9, 10), such as anti-oxidative stress, anti-inflammatory, and anti-aging properties in many disease models. Importantly, it has been reported to confer a promising protective effect against renal I/R injury in a variety of kidney diseases, although its efficacy is debated (11, 12). There were three randomized controlled trials applied resveratrol supplementation in chronic kidney disease patients, and had obtained definite curative effect, it has the potential to become a routine treatment used drug for treating kidney diseases (13–15). To summarize the latest information on the protective effects of RSV, this study conducted a systematic review to provide a deep insight into the efficacy of RSV for renal I/R and discuss the underlying mechanisms.

## Methods

### Search strategy

The following electronic databases were searched from their inception dates to June 2022 for animal studies on the renal protective effects of RSV: PubMed, Embase, Web of Science, and Science Direct. The search terms included “renal ischemia-reperfusion injury,” “renal ischemia/reperfusion injury,” “renal I/R injury,” “renal IR,” “resveratrol,” “RSV,” and “*Polygonum cuspidatum.*” The reference lists of the included articles were also searched to identify additional studies. No language restriction was applied to the search results.

### Study selection

The titles and abstracts of the studies identified by the literature search were screened for eligibility by two independent reviewers (LTY and DRL), full texts were checked only when abstracts itself were not able to reveal the nature of the studies. The filters applied were animal experiment, I/R injury in renal or kidney, and the injury was evaluated by at least one biochemical indicator. The third reviewer (QYL) was consulted if there is any disagreement, who is also responsible for re-check all unextracted studies to make sure no study that fulfilling the inclusion criteria was mistaken abandoned. The potentially relevant articles were then retrieved, and the two reviewers assessed the full texts to determine whether they met the eligibility criteria independently. Disagreements were settled by the third reviewer to reach a consensus.

### Eligibility criteria

#### Types of studies

All controlled studies with RSV administration to experimental animals with renal I/R injury *in vivo* were searched. All clinical case reports and solely *in vitro* experiments were excluded. There was no language, publication date, or publication status restriction.

#### Types of participants

Any laboratory animal exposed to renal I/R injury, regardless of species, strain, gender, or age, was included. The duration of ischemia and reperfusion were also not limited. However, AKI models conducted on transplanted kidneys, folic acid, and genetically modified models were excluded.

#### Types of intervention

Any types of RSV interventions were included, regardless of dosage, dissolvent, formulation, route of administration, administration time, or duration. Animals treated with natural analogs of RSV, extraction of a single active ingredient from RSV, or special copolymers to increase RSV delivery [i.e., NMDAR inhibitor (DAP5)] were not included.

#### Types of comparison

Any types of comparison including empty control or placebo were included. Placebo controls were not limited to physiological saline, absolute ethanol, or DMSO.

#### Type of outcome measure

Indicators of renal function, including blood urea nitrogen (BUN) and serum creatinine (SCr), were considered as primary outcomes and were converted to unified units, regardless of the different detection methods. The biochemical indexes related to oxidative stress were considered secondary outcomes, including malondialdehyde, glutathione and catalase levels. The testing methods or measurement units of malondialdehyde (MDA), glutathione (GSH), and catalase (CAT) were not limited.

### Data extraction

Two reviewers (YDS and ZTT) extracted data from included studies independently, and any discrepancies were resolved by discussion and consensus, then the data were checked by another two reviewers (LTY and DRL). The following information was collected from each piece of literature and summarized: (1) study characteristics, (2) animal characteristics, (3) I/R injury, (4) interventions (i.e., groups, route of administration, dosage, timing, and duration of treatment), and (5) BUN, SCr, MDA and GSH levels. The mean outcome, standard deviation, and the number of animals per group were collected from each study.

The data available only in graphs were re-evaluated with GetData Graph Digitizer 2.24. Furthermore, to increase consistency and avoid the risk of bias, all the data extraction steps were conducted by two reviewers (GJD and ZY) independently. An error range of ≤1% for mean data between the two reviewers was considered acceptable. In case of disagreements, a third investigator (QYL) extracted the data again and made the final decision.

### Quality assessment

The methodological quality was assessed by using the CAMARADES 10-item checklist^1^ : (1) peer-reviewed journal; (2) temperature control; (3) animals were randomly allocated; (4) blind established model; (5) blinded outcome assessment; (6) anesthetics used without marked intrinsic neuroprotective properties; (7) animal model (diabetic, advanced age or hypertensive); (8) calculation of sample size; (9) statement of compliance with animal welfare regulations; (10) possible conflicts of interest (16, 17).

For each item, bias was assessed as a low or high risk of bias, and “unclear” indicated that the risk of bias was not clear. Terms evaluated as low risk were marked with a symbol “+,” which also indicated a quality score. Studies with >5 points were considered high quality.

### Statistical analysis

Outcomes were pooled if they were reported in at least three studies. The data were analyzed separately by Review Manager 5.3 software. The weighted mean difference was used to present outcomes with the same unit, while the standardized mean difference was applied for outcomes with different units. The differences between the RSV and control groups were assessed by comparing the final results of the studies. The heterogeneity of the included studies was assessed by *I*^2^. For all analyses, the fixed effect model was used to analyze the pooled effects when heterogeneity was insignificant (i.e., *p* > 0.1; *I*^2^ ≤ 50%); otherwise, the random effect model was applied. Subgroup analysis was conducted according to predefined variables, including ischemia duration, method of model establishment, route of administration, dosage, total administration time, and risk of bias. Funnel plots were generated to illustrate the potential risk of publication bias.

## Results

### Selection of studies

**FIGURE 1:**
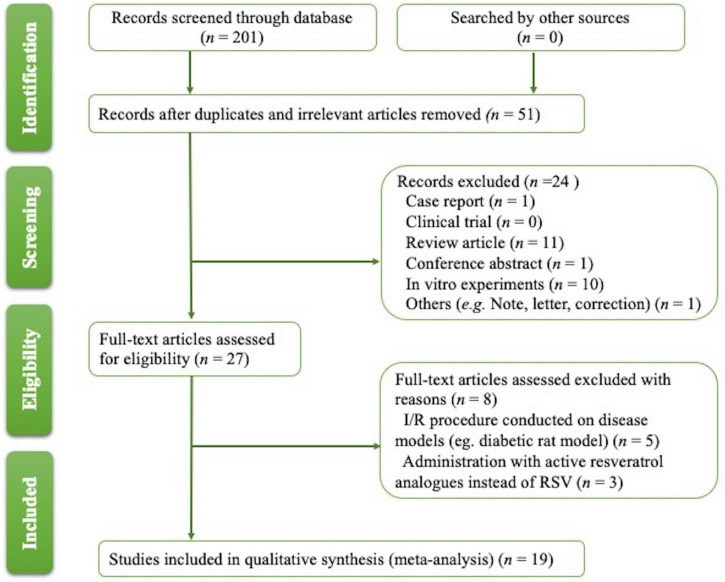
Summary of the literature identification and selection process.

The process for article selection is shown in Figure 1. A total of 201 publications were identified. After removing duplicates and performing title/abstract screening, 31 papers were selected for full-text screening, and 19 met the inclusion criteria (Table 1) (18–36). Eight publications were excluded after reading the full text; six studies were excluded as other disease models (37–42) complicated with I/R were used; three papers were filtered out for using biologically active RSV analogs instead of RSV (43–45).

**TABLE 1:** Characteristics of the studies included in the review.

References	Animals	I/R	No. of animals	Groups	Met	Renal function
Giovannini et al. (22)	Male Wistar rats (200 ± 40 g)	Both renal pedicles were cross-clamped for 40 min, then reperfusion for 24 h	E = 15, G = 15	(1) Sham (A) Sham (B) RSV (C) Vehicle (D) L-NAME (2) I/R (E) I/R + Sham (F) I/R + Vehicle (G) I/R + RSV (H) I/R + L-NAME (I) I/R + L-NAME + RSV	0.23 μg/kg, *i.v.* before ischemia	SCr, U-creatine, TBARS in renal cortex and medulla, nitric oxide formation, and mortality rate
Bertelli et al. (18)	Male Wistar rats (220 ± 20 g)	Both renal pedicles were cross-clamped for 40 min, then reperfusion for 24 h	B = 15, C = 15	(A) Sham (B) I/R (C) RSV + I/R (D) L-NAME + I/R	1 μM *i.v.* 40 min before ischemia	SCr, urinary cGMP, and histological examination
Chander and Chopra (21)	Male Wistar rats (200–250 g)	Occluding both renal pedicles and then reperfusion for different design. IP: three cycles of 2 min of ischemia and 5 min of reperfusion and 45 min of ischemia followed by 24 h of reperfusion	6/6/7/6/8/7/6/6	(A) Sham (B) I/R (C) IP + I/R (D) I/R + RSV (E) IP + I/R + RSV (F) IP + I/R + L-NAME (G) IP + I/R + RSV + L-NAME	RSV (5 mg/kg) by gavage	SCr, BUN, creatinine clearance, TBARS, enzymatic activity of GSH, Catalase, SOD, renal tissue NO, urine NO, and morphologic changes
Saito et al. (24)	Wistar male rats (223–269g)	Right nephrectomy and 1 h clamping of the left renal pedicle, then reperfusion 7 days	6/6/6/6	(A) I/R (B) I/R + 10% ethanol (C) I/R + RSV (D) Sham	4 mg/kg *i.p.* daily from 1 day before ischemia through to 7 days after the operation	SCr, CD86 mRNA, and immunofluorescence staining of CD86 proteins in renal cortex
Chander and Chopra (20)	Male Wistar rats (150–200 g)	Right nephrectomy 7 days before, 45 min of renal ischemia followed by 24 h of reperfusion	8/8/8/8/8/8	(A) Sham (B) UNI (C) UNI + I/R (D) UNI + L-NAME + I/R (E) UNI + RSV + I/R (F) UNI + RSV + L-NAME + I/R	5 mg/kg, *p.o.* followed by I/R	BUN, urea clearance, SCr, creatinine clearance, urine total NO, MDA, GSH, SOD, CAT, and histopathological examination
Sener et al. (25)	Male Wistar albino rats (200–250 g)	Right nephrectomy, then the left renal pedicle was occluded for 45 min, followed by reperfusion for 6 h	8/8/8/8	(A) Sham (B) RSV (C) I/R + Saline (D) I/R + RSV	30 mg/kg *i.p.* 30 min prior to ischemia and immediately before the reperfusion period	BUN, Cr, MPO, MDA, GSH, collagen content in renal tissue, TNF-α, LDH, and lucigenin
Budak et al. (19)	52 New Zealand white rabbits (mean weight 3.6 ± 0.4 kg)	The infrarenal aorta clamped for 30 min reperfusion for 30 min	6/14/8/5/5/8/6	(A) I/R (B) I/R + RSV (I) (C) I/R + RSV (R) (D) I/R + L-NAME (I) (E) I/R + L-NAME (R) (F) I/R + RSV (I) + L-NAME (R) (G) I/R + L-NAME (I) + RSV (R)	1 mg/kg *i.v*. in the first 5 min of ischemia and/or reperfusion. [Notes of drugs were according to the administration period of resveratrol and/or L-NAME (ischemia or reperfusion)]	BUN, SCr, CKMB, AST, ALT, LDH, GGT, amylase, lipase, histopathological assessment (myocardial biopsies, lung biopsies, liver biopsies, renal biopsies, and bowel biopsies)
Saito et al. (23)	Male SD rats at 6 weeks of age	Both renal pedicles clamped for 30 min, followed by reperfusion for 48 h	4–6 rats in each group	(A) Sham (B) I/R (C) I/R + AST-120 (D) I/R + RSV (E) I/R + Quercetin (F) I/R + Sulforaphane	5 mg/kg, *p.o*, 24 and 1 h before and 24 h after renal I/R	SCr, BUN, serum IS conc., renal IS conc., serum MDA, urinary Kim-1 excretion, Nrf2, OAT1, and OAT3
Xiao et al. (31)	Eight week old male C57BL/6 mice	Both renal pedicles clamped for 30 min, followed by reperfusion for 4 days	8/8/8	(A) Sham (B) I/R (C) I/R + RSV	Daily 20 mg/kg gavage for 6 weeks	BUN, SCr, α-SMA, COL1A1, and E-cadherin
Bienholz et al. (27)	Male SD rats (400–470 g)	Bilateral renal clamping for 45 min, followed by reperfusion for 3 h	8/8/8/8/8	(A) Sham (B) I/R (C) Sham + RSV (D) I/R + RSV_*high*_ (E) I/R + RSV_*low*_	RSV_*high*_: 1.19 mg/kg (0.28 mg/kg/h within 1 day) RSV_*low*_: 0.238 mg/kg (0.056 mg/kg/h within 1 day)	Urine output, pCrea, pCysC, SIRT1, IL-6, and TNF-α
Erkasap et al. (29)	3–4 months male SD rats (200–250 g)	Left renal artery clamped for 60 min, followed by reperfusion for 60 min	8/8/8/8/8	(A) Sham (B) I/R (C) I/R + leptin (D) I/R + RSV (E) I/R + leptin + RSV	20 mg/kg, oral gavage, 14 days before the I/R procedure	TNF-α, TNF-α R1, NF-κB, SIRT-1, STAT1 and STAT3 mRNA levels, and caspase 3 protein levels
Xu et al. (35)	8–10 weeks Male SD rats (290–300 g)	Clamping both renal vessels for 30 min followed by reperfusion for 24 h	5/5/5/5/5/5	(A) Sham (B) I/R (C) I/R + DAP5 (D) I/R + RSV (E) I/R + RSV + DAP5 (F) I/R + RSV + DAP5-NP	2 mg/kg *i.v.* 3 h before I/R surgery	Cr, BUN, apoptosis rate, Caspase-3, pathological damage score, SOD, ROS, MDA, p-DAPK, p-CaMK, p-AKT, TNF-α, IL-1β, and IL-6 p-IκBα
Li et al. (33)	40 male SD (200–220 g)	Right kidney removed, left kidney pedicle blocked for 45 min, followed by reperfusion for 1 week	10/10/10/10	(A) Sham (B) I/R (C) I/R + RSV (D) I/R + RSV-NPs	40 μg/g *i.v.* shortly after I/R	Histological examination, BUN, SCr, CRP, apoptosis rate, apoptosis of lymphocyte, MDA, GSH-PX, NGAL, KIM-1, IGFBP2, caspase-3, Bcl-2, and Bax
Li et al. (34)	Male SD rats (200–250 g)	Clamped left renal artery for 60 min followed by right nephrectomy, reperfusion for 24 h	5/5/5/5	(A) Sham (B) I/R (C) RSV (D) I/R + RSV	0.23 μg/kg *i.g.* 30 min before I/R	SCr, BUN, MDA, SOD, HE staining scores, number of infiltrated lymphocytes, IFN-γ, IL-6, TNF-α, IL-10, MMP-13, IFN-γ, IL-6, TNF-α, IL-10, and TLR4/NF-κB signaling pathway
Baltaci et al. (26)	Wistar albino adult male rats (250–260 g)	Left kidney ischemia for 60 min, followed by reperfusion for 60 min	8/10/10/10/10	(A) Sham (B) I (C) I/R (D) I + RSV (E) I/R + RSV	60 mg/kg/day, 3 weeks before ischemia and reperfusion period	Plasma MDA, erythrocyte GSH, and renal MDA, and GSH
Buys-Gonçalves et al. (28)	9 weeks male Wistar rats	Left renal artery and vein clamped for 60 min, then reperfusion for 4 weeks	39	(A) Sham (B) Sham + RSV (C) I/R (D) I/R + RSV	30 mg/kg *i.p.* 60 min before the surgical procedure	Urea, SCr, kidney weight, kidney volume, cortical volume, cortex-non-cortex areas ratio, Vv[Glom], VWGV, and number of glomeruli
Gong et al. (30)	Male SD rats (200 ± 20 g)	Bilateral renal pedicles clamped for 30 min, followed by reperfusion for 1, 3, and 7 days	5/5/5/5	(A) Sham (B) I/R (C) I/R + RSV	30 mg/kg, *i.p.* once a day for 3 days before ischemia, and 30 min before surgery	BUN, SCr, tissue cleaved caspase3, tubular injury score, LC3II/I ratio, and beclin1
Hemsinli et al. (36)	3–4 months male SD rats (257 ± 38 g)	Exposed to 60 min shock, followed by ischemia for 60 min and reperfusion for 120 min	8/8/8/8	(A) Sham (B) I/R (C) Sham (D) I/R + RSV	10 mg/kg *i.p.* 15 min prior to ischemia and immediately before reperfusion	BUN, SCr, MDA, GSH, CAT, NO, apoptotic renal tubular cell numbers, caspase-3 levels, and tubular necrosis scores
Ye et al. (32)	40 SD rats aged 8–10 weeks and weighing 210–250 g	Right kidney removed, left renal hilum ligated for 30 min, followed by reperfusion for 24 h	10/10/10/10	(A) Sham (B) I/R (C) I/R + CMC-N (D) I/R + RSV	25 mg/kg via gavage for 2 weeks before the operation	SCr, BUN, SOD, CAT, GSH-PX, cleaved caspase-3, SIRT1, PGC-1α, NRF1, TFAM, and mitochondrial biogenesis-related proteins

AST, aspartate aminotransferase; ALT, alanine aminotransferase; α-SMA, α-smooth muscle actin; Bax, BCL2-associated X; Bcl-2, B-cell lymphoma-2; BUN, blood urea nitrogen; CRP, C-reactive protein; CaMK, calmodulin-dependent protein kinase; CAT, catalase; CKMB, creatine kinase myocardial band; COL1A1, collagen type I alpha 1 chain; CysC, cystatin C; DAPK, death-associated protein kinase; GSH, glutathione; GSH-Px, glutathione peroxidase; GGT, gamma-glutamyl transpeptidase; KIM-1, kidney injury molecule-1; IFN-γ, Interferon-γ; IGFBP2, Insulin Like Growth Factor Binding Protein 2; IKK, inhibitor of nuclear factor kappa kinase; IκB, inhibitor of NF-κB; IL-1, interleukin-1; IL-6, interleukin-6; LDH, lactate dehydrogenase; MDA, malondialdehyde; MMP-13, matrix metallopeptidase 13; MPO, myeloperoxidase; Myd88, myeloid differentiation factor 88; NF-κB, nuclear factor-kappa B; NGAL, neutrophil gelatinase-associated lipocalin; NO, nitric oxide; NRF, nuclear respiratory factor; OAT3, organic anion transporter 3 polypeptide A; PGC-1α, peroxisome proliferator-activated receptor gamma co-activator 1 alpha; PKB = AKT, protein kinase B; SCr, serum creatinine; SD, Sprague-Dawley; SIRT-1, sirtuin-1; SOD, superoxide dismutase; STAT1, signal transducers and activators of transcription 1; STAT3, signal transducers and activators of transcription 3; TBARS, thiobarbituric acid reactive substances; TFAM, mitochondrial transcription factor A; TLR4, toll-like receptor 4; TNF-α, tumor necrosis factor-α.

### Characteristics of the studies included in the meta-analysis

Most of the selected studies were conducted in Turkey (*n* = 5) and China (*n* = 6). The other studies were performed in Italy (*n* = 2), Japan (*n* = 2), India (*n* = 2), Brazil (*n* = 1), and Germany (*n* = 1). In total, 18 studies were conducted on male rats (9 studies used SD rats, 7 used Wistar rats, 1 used C57BL/6 mice, and the last study did not specify), and only one study was conducted on rabbits. The sample size ranged from 5 to 15. The duration of ischemia was generally 30–60 min, while the reperfusion time varied from 30 min to 7 days. In addition, 3 studies adopted a unilateral renal I/R injury model (26, 28, 29), while a nephrectomy with contralateral clamping was performed in 6 of the remaining studies (21, 24, 25, 32–34), and eight studies were conducted on both sides (19, 21–23, 27, 30, 31, 35). The RSV dosage was <5 mg/kg in six studies (19, 22, 24, 27, 34, 35), 5–10 mg/kg in three studies (20, 21, 23), and ≥10 mg/kg in six studies (25, 26, 28–33, 36). Most studies used oral administration of RSV, while a few performed intravenous injection (18, 19, 22, 27, 33, 35) and intraperitoneal injection (24, 25, 28, 30, 36). All studies reached a consensus after discussing with a third reviewer to settle disagreements.

### Risk of bias within studies

Two independent reviewers assessed the risk of bias for all studies with a quadratic-weighted kappa value (Kw) of 0.846 and achieved 100% after consensus (Table 2). All 19 studies are peer-reviewed publications with appropriate animal models. Most studies complied with animal welfare regulations, and the potential conflict of interests or study funding were clearly stated, except for one (31). 42.1% (8/19) of the studies did not elaborate on temperature control (19, 20, 23, 24, 28, 29, 31, 36), while random allocation to the treatment or control group was absent in 47.4% (9/19) of the studies (18, 19, 21, 23–25, 27, 31, 35). 31.6% (6/19) publications assessed the outcomes with blinded methods (20, 21, 27, 34–36). 84.2% (16/19) of included studies were considered high-quality studies, which suggested the studies met high standards overall (16, 20–23, 25–36). However, they shared common methodological limitations as none of them reported sample size calculation, and all of them lacked masked induction of ischemia.

**TABLE 2:** The research quality of included studies.

References	1	2	3	4	5	6	7	8	9	10	Score
Giovannini et al. (22)	+	+	+	?	?	+	+	?	+	+	7
Bertelli et al. (18)	+	+	?	?	?	+	+	?	+	?	5
Chander and Chopra (21)	+	+	?	?	+	+	+	?	+	+	7
Saito et al. (24)	+	?	?	?	?	+	+	?	+	?	4
Chander and Chopra (20)	+	?	+	?	+	+	+	?	+	+	7
Sener et al. (25)	+	+	?	?	?	+	+	?	+	?	5
Budak et al. (19)	+	?	?	?	?	+	+	?	+	?	4
Saito et al. (23)	+	?	?	?	?	+	+	?	+	+	5
Xiao et al. (31)	+	?	?	?	?	+	+	?	?	+	4
Bienholz et al. (27)	+	+	?	?	+	+	+	?	+	+	7
Erkasap et al. (29)	+	?	+	?	?	+	+	?	+	?	5
Xu et al. (35)	+	+	?	?	+	+	+	?	+	+	7
Li et al. (33)	+	+	+	?	?	+	+	?	+	+	7
Li et al. (34)	+	+	+	?	+	+	+	?	+	+	8
Baltaci et al. (26)	+	+	+	?	?	+	+	?	+	+	7
Buys-Gonçalves et al. (28)	+	?	+	?	?	+	+	?	+	+	6
Gong et al. (30)	+	+	+	?	?	+	+	?	+	+	7
Hemsinli et al. (36)	+	?	+	?	+	+	+	?	+	+	7
Ye et al. (32)	+	+	+	?	?	+	+	?	+	+	7

Studies fulfilling the criteria of the following: (1) peer reviewed publication; (2) control of temperature; (3) random allocation to treatment or control; (4) blinded induction of model; (5) blinded assessment of outcome; (6) use of anesthetic without significant intrinsic vascular protection activity; (7) appropriate animal model (aged, diabetic, or hypertensive); (8) sample size calculation; (9) compliance with animal welfare regulations; (10) statement of potential conflict of interests. “+” represents for certain criteria was described or reported in the study, while “?” means unclear or not mentioned.

### Renal function index

#### SCr

**FIGURE 2:**
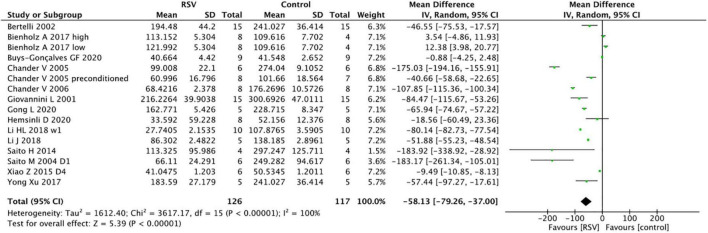
Forest plot for the effects of resveratrol on serum creatinine.

The pooled analysis indicated that RSV significantly decreased SCr levels compared with the control group (16 studies, *n* = 243, WMD = −58.13, 95% CI = −79.26 to −37.00, *p* < 0.00001) (Figure 2). In the subgroup analysis, RSV administration in the dose range of 5−10 mg/kg yielded the highest SCr clearance (four studies, *n* = 51, WMD = −114.86, 95% CI = −171.44 to −58.27, *p* < 0.00001) compared to the higher dose (six studies, *n* = 96, WMD = −40.31, 95% CI = −73.89 to −6.73, *p* = 0.2) and lower dose groups (six studies, *n* = 86, WMD = −48.3, 95% CI = −83.54 to −13.07, *p* = 0.07). RSV provided protective effect in the bilateral renal clamping model (13 studies, *n* = 193, WMD = −61.97, 95% CI = −86.38 to −37.55, *p* < 0.00001), while the sample size was too small to draw conclusions in unilateral renal clamping model (2 studies, *n* = 34, WMD = −18.79, 95% CI = −21.87 to −15.72, *p* < 0.00001). No significant difference was observed among the diverse routes of administration (*p* = 0.41). The ischemia duration, total administration time and risk of bias level also had no impact on the ability of RSV to clear creatinine (Table 3).

**TABLE 3:** Subgroup analysis of pooled estimates of SCr with different administration of RSV.

Subgroup	No. of studies	Sample size	WMD (95% CI)	*P*-value
**Ischemia duration**				
<45 min	6	50/50	−57.36 [−93.15, −21.56]	=0.002
>45 min	10	76/67	−59.09 [−87.74, −30.45]	<0.0001
**Modeling method**				
Unilateral renal clamping	2	17/17	−54.31 [−159.13, 50.51]	=0.31
Bilateral renal clamping	13	101/92	−61.97 [−86.38, −37.55]	<0.00001
**Route of administration**				
Orally administered (*p.o.*)	7	47/46	−78.16 [−111.86, −44.46]	<0.00001
Intraperitoneal (*i.p.*)	4	28/28	−55.22 [−104.87, −5.58]	=0.03
Intravenous (*i.v.*)	6	61/53	−41.35 [−88.86, 6.15]	=0.09
**Dosage**				
<5 mg/kg	6	47/39	−48.3 [−83.54, −13.07]	=0.007
>5 mg/kg, <10 mg/kg	4	26/25	−114.86 [−171.44, −58.27]	<0.00001
≥10 mg/kg	6	48/48	−40.31 [−73.89, −6.73]	=0.02
**Time of administration**				
≥24 h	5	31/31	−73.05 [−114.91, −31.18]	=0.0006
<24 h	12	90/81	−54.52 [−81.39, −27.65]	<0.0001
**Risk of bias**				
>5	13	105/96	−56.11 [−79.98, −32.44]	<0.00001
≤5	4	31/31	−77.16 [−135.84, −18.48]	=0.01

RSV, resveratrol; SCr, serum creatinine; WMD, weighted mean difference; CI, confidence interval.

#### BUN

**FIGURE 3:**
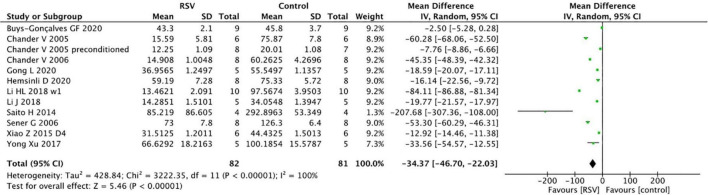
Forest plot for the effects of resveratrol on blood urea nitrogen.

The BUN levels were reported in 13 studies from 12 papers, and the pooled analysis indicated that RSV significantly decreased BUN levels compared to the control group (*n* = 163, WMD = −34.37, 95% CI = −46.70 to −22.03, *p* < 0.00001) (Figure 3). Similar to the SCr results, the subgroup analysis of the optimal dosage to reduce BUN levels was 5–10 mg/kg (four studies, *n* = 51, WMD = −50.12, 95% CI = −80.99 to −19.24, *p* = 0.001), which yielded superior results compared to the higher dose of ≥10 mg/kg (seven studies, *n* = 112, WMD = −27.08, 95% CI = −43.50 to −10.66, *p* = 0.0001) and lower dose of <5 mg/kg (two studies, *n* = 20, WMD = −22.53, 95% CI = −33.33.05 to −11.72, *p* <0.0001). Reduction of BUN levels in bilateral renal clamping model (nine studies, *n* = 113, WMD = −39.36, 95% CI = −54.04 to −24.67, *p* < 0.00001) is obvious, while reduction of BUN in unilateral renal clamping model is unclear (two studies, *n* = 34, WMD = −23.92, 95% CI = −65.92 to 18.07, *p* = 0.26). In terms of BUN levels, ischemic models with longer duration (≥45 min) (nine studies, *n* = 135, WMD = −33.47, 95% CI = −49.03 to −17.91, *p* < 0.00001) benefited more from RSV administration than models with shorter ischemia duration (five studies, *n* = 60, WMD = −16.12, 95% CI = −26.2 to −6.04, *p* = 0.004). However, the prolonged total administration time of RSV (four studies, *n* = 50, WMD = −13.54, 95% CI = −24.29 to −2.79, *p* < 0.00001) did not provide additional protection. The studies did not show significant differences among the administration routes of RSV and different degrees of bias (Table 4).

**TABLE 4:** Subgroup analysis of pooled estimates of BUN with different administration of RSV.

Subgroup	No. of studies	Sample size	WMD (95% CI)	*P*-value
**Ischemia duration**				
<45 min	5	30/30	−16.12 [−26.20, −6.04]	=0.002
≥45 min	9	68/67	−33.47 [−49.03, −17.91]	<0.00001
**Method of model establishment**				
Unilateral renal clamping	2	17/17	−23.92 [−65.92, 18.07]	=0.26
Bilateral renal clamping	9	57/56	−39.36 [−54.04, −24.67]	<0.00001
**Route of administration**				
Orally administered (*p.o.*)	7	47/46	−25.56 [−34.74, −16.37.42]	<0.00001
Intravenous (*i.v.*)	2	15/15	−59.95 [−109.44, −10.46]	=0.02
Intraperitoneal (*i.p.*)	4	30/30	−22.30 [−36.36, −8.24]	=0.002
**Dosage**				
≥10 mg/kg	7	56/56	−27.08 [−43.50, −10.66]	=0.001
≥5 mg/kg, <10 mg/kg	4	26/25	−50.12 [−80.99, −19.24]	=0.001
<5 mg/kg	2	10/10	−22.53 [−33.33, −11.72]	<0.0001
**Total administration time**				
≥24 h	4	25/25	−13.54 [−24.29, −2.97]	=0.01
<24 h	9	67/66	−35.84 [−54.34, −17.33]	=0.0001
**Risk of bias**				
>5	10	74/73	−28.83 [−40.99, −16.68]	<0.0001
≤5	3	18/18	−52.98 [−92.11, −13.85]	=0.008

RSV, resveratrol; BUN, blood urea nitrogen; WMD, weighted mean difference; CI, confidence interval.

### Important mechanism indicator

**FIGURE 4:**
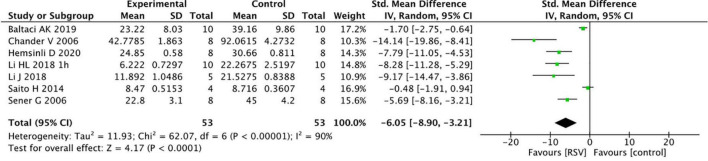
Forest plot for the effects of resveratrol on malondialdehyde.

**FIGURE 5:**
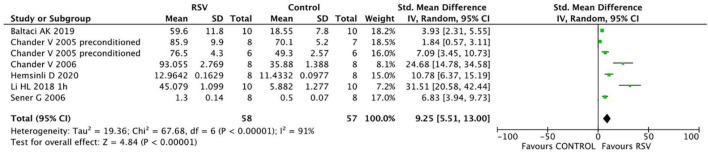
Forest plot for the effects of resveratrol on glutathione.

**FIGURE 6:**
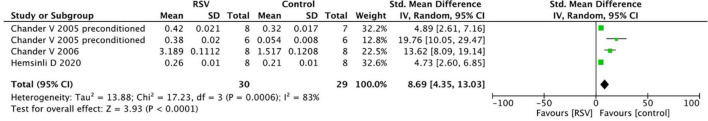
Forest plot for the effects of resveratrol on catalase.

Common oxidative stress indicators related to renal protective mechanisms were extracted from the selected articles. The MDA levels were significantly reduced by RSV (seven studies, *n* = 106, SMD = −6.00, 95% CI = −6.05, 95% CI = −8.90 to −3.21, *p* < 0.0001) compared with the control group (Figure 4). The GSH levels were notably improved after RSV administration (seven studies, *n* = 115, SMD = 9.25, 95% CI = 5.51–13.00, *p* < 0.00001) (Figure 5), as well as the CAT levels (four studies, *n* = 59, SMD = 8.69; 95% CI = 4.35–13.03, *p* < 0.0001) (Figure 6).

### Publication bias

**FIGURE 7:**
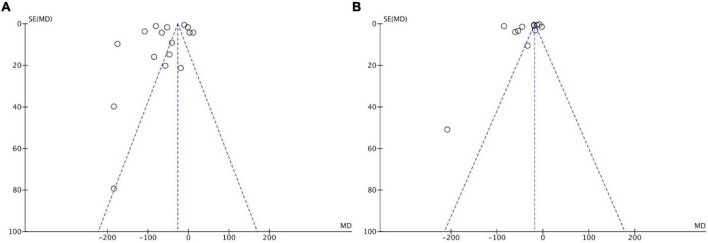
Funnel plots on publication bias for serum creatinine **(A)** and blood urea nitrogen **(B)**.

Funnel plots of the studies, including BUN and SCr levels, were used to assess publication bias (Figure 7). Asymmetrical funnel plots for the meta-analysis of BUN and SCr were obtained, suggesting a certain risk of publication bias.

## Discussion

### Summary of evidence

This is the first preclinical meta-analysis to estimate the efficacy and possible mechanism of RSV in the treatment of renal I/R injury. 84.21% (16/19) of enrolled articles were considered under prudent design and assessment. This meta-analysis indicated that RSV administration significantly improves renal function (lowering the SCr and BUN levels) by alleviating oxidative stress (lowering MDA and promoting GSH and CAT). The optimal protective effect was achieved at the dosage between 5 and 10 mg/kg, while higher doses or prolonged utilization did not yield additional benefits. Other influence factors, such as the timing of administration, duration of ischemia, unilateral or bilateral I/R injury, as well as methodological quality, did not significantly impact the effects of RSV.

### The possible mechanism for the effect of RSV in renal I/R injury

**FIGURE 8:**
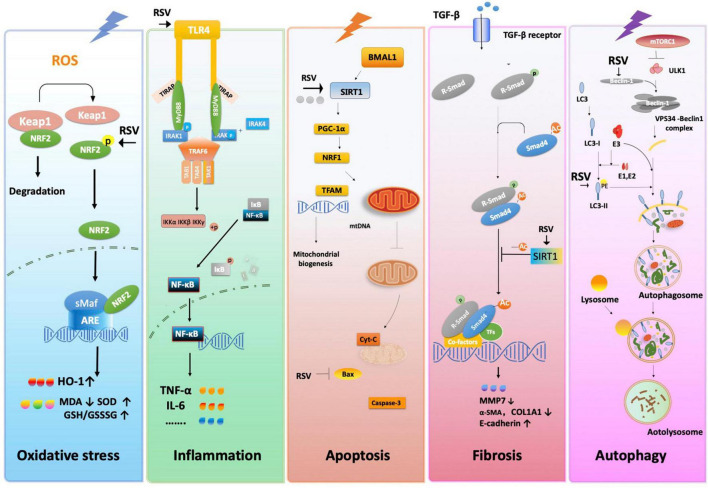
The proposed molecular and cellular mechanism of the protective effect of resveratrol for renal I/R injury. The figure was created based on the data of the studies. ARE, antioxidant response element; α-SMA, α-smooth muscle actin; Bax, BCL2-associated X; BMAL1, gene BMAL1; COL1A1, collagen type I alpha 1 chain; Cyt-C, cytochrome C; GCL, glutamate-cysteine ligase; GSR, GSH *S*-reductase; HO-1, heme oxygenase-1; Iκκ, inhibitor of nuclear factor kappa kinase; IκB, inhibitor of NF-κB; IL-6, interleukin-6; IRAK1, interleukin-1 receptor-associated kinases; Keap1, kelch-like ECH-associated protein 1; MMP-13, matrix metallopeptidase 7; mtDNA, mitochondrial DNA; mTORC1, mTOR complex 1; Myd88, myeloid differentiation factor 88; NF-κB, nuclear factor-kappa B; Nrf2, nuclear factor E2-related factor 2; PE, phosphatidylethanolamine; PGC-1α, peroxisome proliferator-activated receptor gamma co-activator 1 alpha; ROS, reactive oxygen species; R-SMAD, regulatory SMADs; RSV, resveratrol; SIRT-1, sirtuin-1; sMaf, small Maf; TAB1, TGF-β- activated protein kinase 1; TAK1, transforming growth factor beta-activated kinase1; TFAM, mitochondrial transcription factor A; TFs, transcription factors; TGF-β1, transforming growth factor-β; TIRAP, toll interleukin 1 receptor domain containing adapter protein; TLR4, toll-like receptor 4; TNF-α, tumor necrosis factor-α; TRAF6, tumor necrosis factor receptor-associated factor 6.

Various hemodynamic and physiological factors are involved in AKI pathophysiology, including cell injury and subsequent inflammatory responses, oxidative stress, the regulation of glomerular blood flow, autophagy and apoptosis. Interactions between these different mechanisms drive renal injury, and the underlying mechanism of RSV likely produces a multi-faceted effect. The reported protective mechanisms of RSV against acute renal I/R injury are summarized below (Table 5 and Figure 8).

**TABLE 5:** The proposed molecular and cellular mechanism of the protective effect of resveratrol for renal I/R injury.

References	Mechanism	Effect
Giovannini et al. (22)	Oxidative stress	Upregulated NO, reduced TBARS levels in cortex and medulla
Bertelli et al. (18)	Oxidative stress, inflammation	Decreased TBARS level, reduced PMNs infiltration and IL-6 urinary excretion, reduced urinary cGMP
Chander and Chopra (21)	Oxidative stress	Reduced TBARS, reserved enzymatic activity of GSH, CAT, SOD, upregulate total NO level
Saito et al. (24)	Endothelial cell dysfunction and T cell costimulatory pathways	Decreased CD86 and CD86, attenuate costimulatory adhesion molecules on the vascular endothelium
Chander and Chopra (20)	Oxidative stress	Improved nitric oxide levels, reduced elevated TBARS levels (MDA), and restored the depleted renal antioxidant enzymes (GSH, CAT, and SOD), attenuated morphological alterations
Sener et al. (25)	Oxidative stress	Decreased renal MDA, preserved GSH, blocked neutrophil infiltration (decreased MPO activity), abolished the increase of collagen content in renal tissue, decrease TNF-α, LDH activity, and the elevations in lucigenin
Budak et al. (19)	Oxidative stress	Decreased I/R scores and corresponding histopathological sample, lower number of PMNLs
Saito et al. (23)	Oxidative stress	Inhibited hepatic SULT activity, suppressed Nrf2, restored OAT1 and OAT3
Xiao et al. (31)	Oxidative stress, antifibrosis	Increased E-cadherin, decreased α-SMA and COL1A1, up-regulated SIRT1 and decreased MMP-7
Bienholz et al. (27)	Oxidative stress	Negative effect on blood pressure, increased TNF-α, while reduce pCysC
Erkasap et al. (29)	Apoptosis	Increased TNF-α, TNF-α R1, NF-κB, increase the activity of JAK/STAT pathway, decreased caspase 3
Xu et al. (35)	Oxidative stress, apoptosis, and inflammation	Reduced ROS, MDA, caspase-3, decreased TNF-α, IL-1β, and IL-6, and p-IκBα
Li et al. (33)	Oxidative stress, and apoptosis	Decreased MDA, Bax and caspase3, increased GSH-Px, Bcl-2
Li et al. (34)	Oxidative stress, inflammatory, mitochondrial stress and apoptosis	Reduced MDA and increased SOD, upregulated Nrf2/ARE/Ho-1 signal pathway, inhibited TLR4/NF-κB signaling pathway, downregulated caspase-3, cleaved caspase-3, caspase-8 and caspase-9
Baltaci et al. (26)	Oxidative stress	Increased GSH and decreased MDA
Buys-Gonçalves et al. (28)	Prevent glomerular loss (no mechanism)	Preserved N[Glom] and Vv[Glom] (unbiased quantitative analysis of the morphometrical aspects of kidney)
Gong et al. (30)	Autophagy and apoptosis	Decreased cleaved caspase-3, increase LC3II/I ratio and upregulate beclin1
Hemsinli et al. (36)	Oxidative stress and apoptosis	Increased GSH, CAT and NO, reduced MDA, caspase-3, apoptotic renal tubular cell numbers, and tubular necrosis scores
Ye et al. (32)	Apoptosis	Increased SIRT1, PGC-1α, NRF1, and TFAM

α-SMA, α-smooth muscle actin; AKT, protein kinase B; Bax, BCL2-associated X; Bcl-2, B-cell lymphoma-2; CAT, catalase; COL1A1, collagen type I alpha 1 chain; CysC, Cystatin C; GSH, glutathione peroxidase; HO-1, hemeoxygenase-1; IFN-γ, interferon-γ; IκB, inhibitor of NF-κB; IKK, inhibitor of nuclear factor kappa kinase; IL-1β, interleukin-1β; IL-6, interleukin-6; MDA, malondialdehyde; JAK, Janus kinase; LDH, lactate dehydrogenase; MMP, matrix metalloproteinase; MPO, myeloperoxidase; MyD88, myeloid differentiation factor 88; NF-κB, nuclear factor-kappa B; NMDA, N-methyl-D-aspartic acid; NO, nitric oxide; OAT, organic anion transporters; PGC-1α, proliferator-activated receptor gamma co-activator 1 alpha; PMNL, poly morphonuclear leukocyte; α-SMA, α-smooth muscle actin; SOD, superoxide dismutase; STAT, signal transducers and activators of transcription; Nrf, nuclear factor E2-related factor; SIRT-1, sirtuin-1;TBARS, thiobarbituric acid reactive substances; TFAM, mitochondrial transcription factor A; TNF-α, tumor necrosis factor-α; TLR 4, toll-like receptor; Vv[Glom], glomerular volumetric density; N[Glom], total number of glomeruli per kidney.

### Protective effect of RSV related to oxidative stress

Oxidative stress is characterized by a high concentration of ROS, which leads to ATP depletion, increasing intracellular Ca^2+^ signals, and activating membrane phospholipid proteases and mitochondrial oxidative phosphorylation (46). The kidney is vulnerable to internal oxidative stress during I/R injury, especially in tubular epithelial cells in the reperfusion phase of I/R.

Our meta-analysis confirms that RSV inhibits MDA and promotes super oxide dismutase (SOD) and CAT. Normally, nuclear factor erythroid 2-related factor 2 (Nrf2) is bound by Kelch-like ECH-associated protein 1 (Keap1), maintaining low levels in the cytoplasm. Conformational changes of Keap1 protect Nrf2 against oxidative stress under I/R. The expression of Hemeoxygenase-1 (HO-1), an antioxidant response elements (ARE)-dependent phase II detoxifying enzyme and antioxidant, is regulated by Nrf2, suggesting that upregulation of Nrf2 is essential for HO-1-mediated cytoprotection against I/R (34, 47). Modulation of Nrf2 and its downstream gene expression finally stimulates the activity of antioxidant enzymes such as SOD, GSH and CAT while downregulating the concentration of MDA. Evidence showed that long-term administration of RSV lowers oxidative stress by activating the transcription factor nuclear factor-E2-related factor-2 (Nrf2), one of the regulators of antioxidant cell defense, and impaired Nrf2-GST activity in the kidney, thus mitigating renal inflammation and injury (48). Besides, RSV was also reported to scavenge ROS directly (49).

### Protective effect of RSV related to inflammation

Cell damage and related molecular products caused by I/R activate the pattern recognition receptors and trigger subsequent expression of inflammation-related genes. Toll-like receptor (TLR) plays an important role in the NF-κB and MAPK pathways, producing inflammatory factors such as TNF-α, Interleukin (IL)-1, IL-17, and IFN-γ. The formation of excessive free radicals and mitochondrial damage also cause complex inflammation reactions. Both of them amplify and prolong cell damage and cell death.

The RSV alleviates innate immunity and inflammatory response by inhibiting the activation of pattern recognition receptors. For example, TLR4 expression was reportedly attenuated after RSV administration (50). RSV also exhibits anti-inflammatory effects through a series of pathways and their downstream gene expression. Qi et al. (51) demonstrated that RSV protects cells from inflammatory responses by blocking the TLR4-MyD88-NF-κB signal pathway. Moreover, RSV inhibits NF-κB activation by suppressing TNF-induced phosphorylation and nuclear translocation of the p65 subunit of NF-κB (52). The activation of Nrf2 also exerts anti-inflammation effects by regulating the TLR4 pathway in various tissues (34, 53).

### Protective effect of RSV related to apoptosis

Apoptosis is a complex pathological process triggered by extrinsic and intrinsic pathways (54). In the intrinsic pathway, accumulating ROS, Ca^2+^ overload, endoplasmic reticulum (ER) stress, and damaged DNA are amplified in I/R injury and are involved in both caspase-related and caspase-independent ways. Mitochondrial imbalance also plays an important role during apoptosis. I/R injury down-regulates the expression of BMAL1/SIRT/PGC-1α and decreases the concentration of NRF1 and TFAM, which inhibits the stability of mtDNA. A decrease in mtDNA inhibits the mitochondrial biogenesis level. Consequently, the mitochondrial homeostasis and increase in oxidative stress result in mitochondrial outer membrane permeabilization (MOMP), which triggers the release of mitochondrial cytochrome C (Cyt C) and activation of the caspase cascade, eventually leading to apoptosis. Meanwhile, Jak/STAT and other pathways may also regulate apoptotic genes in different ways.

Early intervention of I/R-induced renal apoptosis could be effective in preventing renal injury caused by cascade activation (55). Existing studies implied that RSV inhibits apoptosis in renal I/R injury mostly via the intrinsic pathway. For example, RSV has shown anti-apoptotic effects via SIRT1/PGC-1α-mediated mitochondrial protection. As a recognized activator of SIRT1, RSV reverses the release of mitochondrial Cyt C and DIABLO proteins to the cytoplasm, thereby increasing the activity of respiratory chain complex I and III and inspiring mitochondrial membrane potential with anti-apoptotic effects as a result (56).

### Protective effect of RSV related to fibrosis

Different types of kidney diseases subsequently develop into renal fibrosis. Epithelial-mesenchymal transition (EMT) is characterized by increased TGF-β1 and α-SMA protein levels and decreased E-cadherin levels. EMT has been widely accepted as a mechanism in the development of renal fibrosis, especially in tubular epithelial cells. SIRT1 promotes Smad4 deacetylation, thus decreasing MMP7 and E-cadherin by down-regulating ß-catenin/LEF1, consequently inducing EMT and leading to fibrosis.

Evidence indicates that treatment with RSV significantly attenuates extracellular matrix deposition and renal interstitial fibrosis by decreasing the expression of renal cortical ICAM-1, TNF-α and TGF-β, simultaneously upregulating protein expression of fibronectin in a unilateral uretera obstruction model (57). Decreased expressions of fibronectin, collagen IV and TGF-β in glomeruli after RSV administration result in reduced glomerular basement membrane thickness (58). Furthermore, RSV inhibits the TGF-β pathway on MMP7, which attenuates renal injury and fibrosis by inhibiting the EMT process (31). However, the anti-fibrosis function of RSV remains controversial. For instance, Liu et al. (59) reported that the RSV-activated anti-fibrotic or pro-fibrotic effects in kidneys depends on the dosage.

### Protective effect of RSV related to autophagy

Autophagy is a cellular process involving the removal of damaged cytoplasmic components in lysosomes, which are eventually digested into recoverable molecules, while dysregulation of autophagy is implicated in the pathogenesis of various renal diseases (60). DNA damage, energy depletion and the secretion of growth factors affect mTOR complexes1 (mTORC1) through the p53, PI3K/Akt, AMPK, or MAPK/ERK1/2 pathways. The activation of mTORC1 inhibits autophagy by phosphorylating unc-51-like kinase (ULK1), which is the initial step of autophagosomes. It promotes Beclin1 to form the VPS34-Beclin1 complex and induces the formation of PI3K with ATG14. The activated LC3-II forms and extends the membrane of the autophagosome.

The RSV affects many different targets related to autophagy. Some studies reported that RSV regulates the activity of the PI3K/Akt/mTOR pathways by inhibiting Akt phosphorylation at different levels (61). Increased expression of Becn1 and LC3 was observed after RSV treatment, implying the activation of autophagy (30). A similar condition was shown in the diabetic nephropathy model, with increased LC3-II/LC3-I both *in vivo* and *in vitro* (62, 63). Moreover, RSV could inhibit leucine-stimulated mTORC1 activation by promoting mTOR/DEPTOR interaction (64). All the above reveals the multi-faceted mechanism of RSV in the regulation of autophagy.

### Advantages and limitations of this review

The pooled data from the included studies revealed that RSV could be a promising drug for the treatment of renal I/R injury. This review summarizes the possible mechanisms of RSV. A full understanding of the roles and precise mechanisms of RSV will provide deeper insight into the pathological progression of renal I/R injury and the interactions between different renal protection effects. Further research may focus on the rational design of therapeutic drugs. The study was pushed forward at an overall level of each study, which is based on mean and standard deviation instead of individual animal data. Differences exist between the designs of individual studies, and the simple hybrid method inevitably shows its limitations. Besides, all the inconsistencies cannot be analyzed hierarchically. The potential publication bias should also be acknowledged, as evidenced by the asymmetric funnel chart, which may affect the accuracy of our results.

## Conclusion

Our systematic review included data from small animal studies, revealing that RSV enhances renal function in I/R injury models and exhibits antioxidative effects. The underlying mechanism possibly involves reducing ROS, protecting against apoptosis, autophagy, and inflammation and eventually inhibiting the progression of fibrosis. Recent reports revealed that RSV could be a promising drug in the treatment of renal I/R injury. Nevertheless, randomized controlled trials and more intensive animal experiments are required to fully elucidate the mechanism and apply the protective properties of RSV in clinical practice.

## Data availability statement

The original contributions presented in this study are included in the article/supplementary material, further inquiries can be directed to the corresponding author.

## Author contributions

T-YL and R-LD designed the present study. FW and M-HS developed the eligibility criteria, search strategy, risk of bias assessment strategy, and data extraction plan with guidance of D-SY and T-TZ. J-DG and YZ involved in data extraction. Y-LQ solved the disagreements. CW helped to perform the analysis with constructive discussions. All authors wrote the manuscript and contributed to the article and approved the submitted version.

## Footnotes

1
https://www.camarades.de/
